# The role of atria in ventricular fibrillation after continuous-flow left ventricular assist device implantation in ovine model

**DOI:** 10.3389/fcvm.2022.1000352

**Published:** 2023-01-09

**Authors:** Xin-Yi Yu, Jian-Wei Shi, Yan-Sheng Rong, Yuan-Lu Chen, Tian-Wen Liu, Yi-Rui Zang, Ze-An Fu, Jie-Min Zhang, Zhi-Fu Han, Zhi-Gang Liu

**Affiliations:** ^1^Department of Cardiovascular Surgery, TEDA International Cardiovascular Hospital, Chinese Academy of Medical Sciences & Peking Union Medical College, Tianjin, China; ^2^Department of Anesthesiology, TEDA International Cardiovascular Hospital, Chinese Academy of Medical Sciences & Peking Union Medical College, Tianjin, China; ^3^Department of Cardiac Electrophysiology, TEDA International Cardiovascular Hospital, Chinese Academy of Medical Sciences & Peking Union Medical College, Tianjin, China; ^4^Laboratory Animal Center, TEDA International Cardiovascular Hospital, Chinese Academy of Medical Sciences & Peking Union Medical College, Tianjin, China; ^5^Cardiovascular Clinical College of Tianjin Medical University, Tianjin, China; ^6^ROCOR Medical Technology Co., Ltd., Tianjin, China

**Keywords:** left ventricular assist device (LVAD), ventricular fibrillation (VF), atria, arrhythmia, atrial function

## Abstract

**Objectives:**

This study attempted to explore the hemodynamics and potential mechanisms driving pulmonary circulation in status of ventricular fibrillation (VF) following continuous-flow left ventricular assist device (CF-LVAD) implantation.

**Methods:**

An ovine CF-LVAD model was built in small-tailed Han sheep, with the pump speed set as 2,400 rpm. VF was induced following ventricular tachycardia using a temporary pacemaker probe to stimulate the right and left ventricular free walls. The central venous pressure (CVP), pump flow (PF), pulmonary artery flow (PAF) and other major indicators were observed and recorded after VF.

**Results:**

Low-flow systemic and pulmonary circulation could be sustained for 60 min under VF with sinus atrial rhythm after CF-LVAD implantation. The CVP gradually increased. The mean PF declined from 1.80 to 1.20 L/min, and the mean PAF decreased from 1.62 L/min to 0.87 L/min. Under VF with atrial fibrillation, the systemic and pulmonary circulation couldn’t be sustained. The CVP jumped from the 5 mmHg baseline to 12 mmHg, the mean PF rapidly decreased from 3.45 L/min to 0.79 L/min, and the PAF declined from 3.94 L/min to 0.77 L/min.

**Conclusion:**

The atrial rhythm and function might be essential for the circulation maintenance in patients with VF after CF-LVAD implantation.

## 1. Introduction

Mechanical circulatory support (MCS) with continuous-flow left ventricular assist device (CF-LVAD) has been preferably used as a “bridge” device before a heart transplantation and regarded as one of the long-term treatment options for patients with end-stage heart failure. Nonetheless, ventricular arrhythmias (VA) including ventricular fibrillation (VF) which is common in malignant arrhythmias, are frequent after CF-LVAD implantation with an incidence of about 20–60% (1, 2). Under normal physiological status, VF is considered as the main causative factor of sudden cardiac death, as it leads to disappearance of ventricular systolic function and complete loss of cardiac output resulting in severe hemodynamic collapse. Likewise, the VF following CF-LVAD implantation remains to be associated with a risk of low cardiac output or perfusion stop of vital organs and tissues due to the absence of MCS support to the right ventricle (3).

The right heart function is still crucial after CF-LVAD implantation given its role in driving pulmonary blood return to the left heart, which is important for the systemic circulation, even though the CF-LVAD can substitute for the left ventricle to perform the systolic function thereby sustaining the systemic circulation. Theoretically, the VF following CF-LVAD implantation is accompanied by complete loss of the right ventricular squeezing, leading to reductions in CF-LVAD preload and cardiac output, which eventually results in circulatory collapse. To the contrary, no clinical signs of serious circulatory failure are found in VF patients with CF-LVAD in the shorter term, instead some non-specific symptoms such as fatigue, chest tightness or drowsiness (4–7).

Currently, the hemodynamics of VF after CF-LVAD implantation remains elusive. In this study, an acute ovine CF-LVAD model was devised in small tail Han sheep. The systemic and pulmonary circulation was observed and the potential mechanism driving the pulmonary circulation was explored, which may provide possible theoretical explanations for the VF after CF-LVAD implantation.

## 2. Materials and methods

### 2.1. Experimental device

HeartCon Ventricular Assist Device (ROCOR Medical Technology Co. Ltd., Tianjin, China) was applied as the CF-LVAD, with the body made with titanium alloy and a maximum auxiliary flow of 10 L/min (8).

### 2.2. Animal model

Two healthy adult male Small Tail Han sheep, each aged 16- and 18-month and weighed 56 and 58 kg, were used as model A and model B. All the two sheep were purchased from Xi’an Dilepu Biology & Medicine Co., Ltd (SCXK [Shanxi] 2014-004). All the two sheep were housed in separate cages for 2 weeks, and no abnormal clinical signs or blood indicators were observed. All protocols and experimental procedures were approved by the Institutional Animal Care and Use Committee of TEDA International Cardiovascular Hospital. Animal experiments were performed in accordance with the National Institutes of Health guidelines for the Care and Use of Laboratory Animals and the Regulations for the Administration of Affairs Concerning Experimental Animals (2017.03.01 edition) published by the State Council of the People’s Republic of China. At the end of the experiment, the animals were euthanized by injection of high concentrations of potassium chloride under anesthesia.

### 2.3. Surgical preparation and operative procedures

The two sheep were subjected to 24-h fasting and 20-h water deprivation prior to operation. After animal sedation by intramuscular injection of xylazine (1.0 mg/kg), the surgical site was exposed, and the neck skin was prepared. Vascular access was obtained through the great saphenous vein, and an intravenous bolus of propofol (60–200 mg) was injected. Tracheal intubation was then performed with parameters including tidal volume 8–12 ml/kg/min, frequency 12–20 times/min, inspired oxygen concentration 40–100%, positive end-expiratory pressure 5–10 mmHg (1 mmHg = 0.133 kPa) and sevoflurane inhalation 2–4%. A nasogastric tube was inserted for decompression. The arterial puncture was achieved through the left ear to obtain arterial access, and central venous access was obtained via puncture of the left internal jugular vein. Anesthesia was maintained by continuous infusion of dexmedetomidine (0.5–1 mg/kg/h) and succinylcholine (50–100 mg/h).

A thoracotomy was performed at the left fifth intercostal space, and a lidocaine drip (2 mg/min) was initiated to prevent arrhythmias. The pericardium was incised from the apex to the pulmonary artery, and the heart was suspended in a pericardial cradle. The site for pump placement was determined according to the apex position. After obtaining a whole-blood clotting time greater than 450s by intravenous bolus injection of 1.0 mg/kg heparin, the descending thoracic aorta was dissected for outflow graft anastomosis. A partial occlusion clamp was applied, and the pump’s 10-mm outflow graft was sewn end-to-side to the descending aorta with 4-0 Prolene sutures. Hemostasis of the anastomotic site was obtained to reduce the effect of excessive bleeding on capacity loading. The sewing ring was attached to the left ventricular apex with 8 interrupted sutures. The left pleural cavity was filled with warm normal saline. A cruciform incision was made on the beating heart. Then a ventricular punch was advanced through the ventricular sewing ring, the left ventricular core was removed, and the pump was inserted into the left ventricular cavity and secured. The pump was started at 2,000 rpm; then, the artificial blood vessel was opened after full gas exhausting. In the context of circulatory stability, a temporary pacemaker electrode was implanted in the free wall of the right ventricle and left ventricle, respectively.

### 2.4. Research methods

The pump speed was adjusted to 2,400 rpm, and baseline data were recorded after stable vital signs and circulatory stability were obtained. The ventricular rate was controlled by pacing, with the initial rate as 180 beats/min at an increasing rate of 20 beats/min. Rates at 180 beats/min, 200 times/min, 220 times/min and above were recorded and the corresponding circulatory conditions were studied.

A multi-channel physiological recorder (MP150 Biopac, USA) was used to record the heart rate (HR), aortic pressure (AOP) (the measurement point was aortic root), central venous pressure (CVP) (right atrial pressure here), left atrial pressure (LAP) and left ventricular pressure (LVP) etc. A double-channel ultrasonic flowmeter (Transonic, USA) was operated to obtain real-time pump flow (PF) (the measurement point was pump outflow graft) and pulmonary arterial flow (PAF). A blood gas analyzer (IL-1430, USA) was applied to perform arterial blood gas (ABG) analysis every 10–15 min. A color doppler ultrasound (Philips5500, Netherlands) was used to calculate right/left ventricular ejection fraction (RVEF/LVEF) and assess aortic valve function by cardiac surface ultrasound every 10–15 min.

## 3. Results

### 3.1. Baseline data of sheep with CF-LVAD

The baseline pump speed of both model A and model B was 2,400 rpm. Supported by CF-LVAD, the LVEF and RVEF respectively were 65 and 48% in model A while 72 and 64% in model B; the mean PF and PAF respectively were 1.80 and 1.62 L/min in model A while 3.54 and 3.94 L/min in model B; the CVP was 6 mmHg in model A and 5 mmHg in model B (Supplementary Figures 1A, B). The baseline ABG results of model A and model B are exhibited in Supplementary Table 1.

### 3.2. VT after CF-LVAD implantation

**FIGURE 1:**
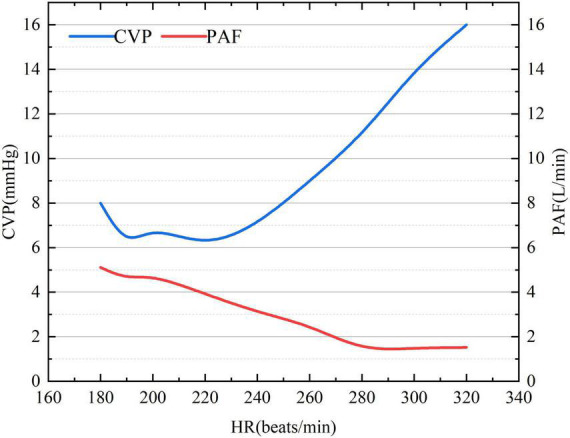
The association of ventricular rate with CVP and PAF under VT after CF-LVAD implantation. CVP, central venous pressure (right atrial pressure here); PAF, pulmonary artery flow.

Under ventricular tachycardia (VT) following CF-LVAD implantation, CVP showed a gradual upward trend while PAF exhibited a downward trend with the increase in ventricular rate (Figure 1). When the ventricular rate reached 280 beats/min, the rate of PAF decline began to gradually slow down. The PAF and CVP basically changed reversely during the whole process.

The cardiac electrical activity, PF, PAF and atrial/ventricular pressure of the animal models are described in Supplementary Figure 2. With the increase in ventricular rate, right atrial pressure (RAP) gradually increased while LAP was relatively stable. In addition, RVP, LVP, PF, and PAF reduced with the increase in ventricular rate.

### 3.3. VF after CF-LVAD implantation

**FIGURE 2:**
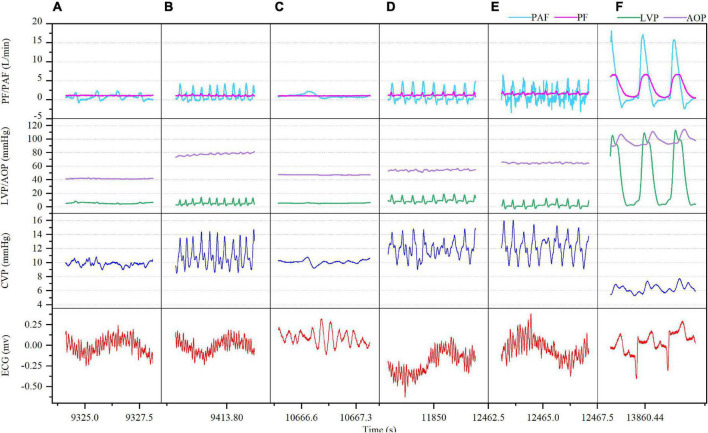
Ventricular fibrillation (VF) with sinus rhythm after CF-LVAD implantation (Model A). **(A)** VF 5 min; **(B)** VF 10 min; **(C)** VF 30 min; **(D)** VF 50 min; **(E)** VF 60 min; **(F)** after defibrillation. PF, pump flow; PAF, pulmonary artery flow; LVP, left ventricular pressure; AOP, aortic pressure.

Ventricular fibrillation was induced in both the model A and model B when the ventricular rate reached 320 beats/min, while there was distinct difference. In model A, VF occurred with sinus atrial rhythm and is accompanied by gradually increased CVP (from 6 mmHg baseline to 10 mmHg at 10 min, 11 mmHg at 30 min and 12 mmHg at 60 min) and waveform amplitude (from 3 mmHg baseline to 7 mmHg at 60 min) with increasing duration. Both LVP and AOP were lower than the normal baseline after VF. Both mean PF and PAF went down while were kept at 1.20 and 0.87 L/min, respectively, during the whole process of VF. The cardiac electrical activity, PF, PAF, CVP, and LVP/AOP after VF were detailed in Figure 2. The ABG results at each time point were shown in Supplementary Table 2. LVEF was always 0 while RVEF was kept at around 36%. At 60 min of VF, sinus rhythm was successfully restored by defibrillation (25 J) (Figure 2F), PF, PAF, LVP, and AOP restored to baseline or slightly higher levels, CVP declined to baseline and the waveform amplitude decreased (Figure 2F).

**FIGURE 3:**
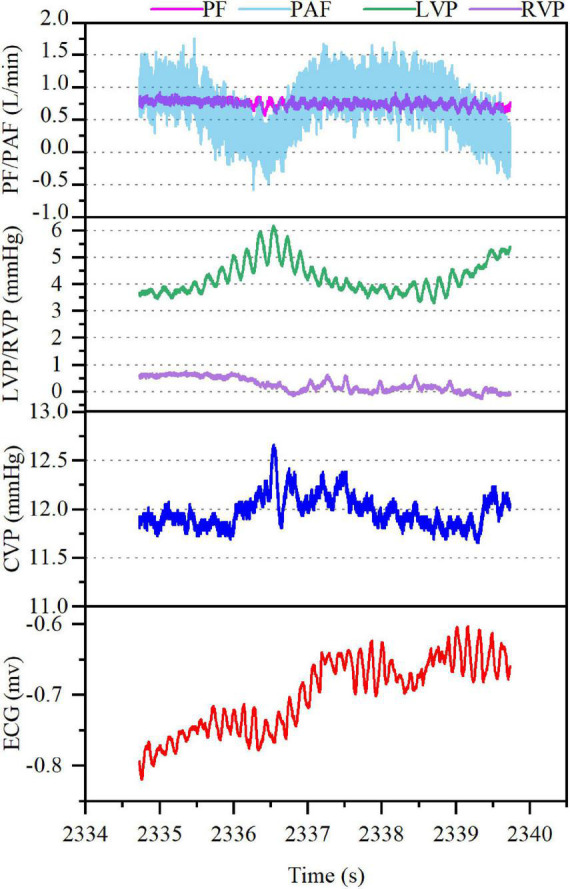
Ventricular fibrillation (VF) with atrial fibrillation after CF-LVAD implantation (Model B). PF, pump flow; PAF, pulmonary artery flow; LVP, left ventricular pressure; RVP, right ventricular pressure.

In model B, VF occurred with AF, accompanied by rapid increase in the mean CVP (from 5 mmHg baseline to 12 mmHg) along with irregular waveform and reduced amplitude. In the meantime, the mean PF (3.54–0.79 L/min) and PAF (3.94–0.77 L/min) declined rapidly with the loss of regular waveform. The LVP and RVP also dropped rapidly, and the RVP fell to nearly 0 mmHg. The cardiac electrical activity, PF, PAF, and LVP/RVP after VF were detailed in Figure 3. The data of ABG were listed in Supplementary Table 3. Circulation of the model B could not be sustained and administration of drugs such as norepinephrine (NE) was ineffective. Besides, immediate defibrillation at 25–50 J failed to restore the sinus rhythm. Both the LVEF and RVEF were 0 on cardiac ultrasound.

## 4. Discussion

Ventricular tachycardia commonly occurs with hemodynamic disorders, together with accelerated ventricular rate and dramatically shortened period of ventricular diastole with deceased filling pressures, resulting in cardiac output reduction. VF is the major cause of sudden cardiac death immediately followed by severe hemodynamic disorders, systemic and pulmonary circulation arrest, and significantly depressed myocardial contractility caused by hypoxia and acidosis. CF-LVAD is mainly developed as an additional pump to the left ventricle to maintain the pumping function. Meanwhile, the native right ventricle remains important for maintaining the normal function of CF-LVAD, as it is responsible for complete pulmonary circulation, which is essential for the maintenance of hemodynamics and provides sufficient preload for CF-LVAD. Theoretically, VF occurs with the stop of native right ventricular contraction instantly followed by pulmonary circulation arrest, resulting in CF-LVAD dysfunction due to insufficient preload. However, patients with CF-LVAD support can survive with less severe clinical symptoms during onset VF, and in short period there may be no serious signs of hemodynamic failure (4–7, 9, 10).

In this study, a CF-LVAD was implanted in small tail Han sheep and VF was induced by increasing ventricular rate using a temporary pacemaker. Our observational study demonstrate that both the model A and model B exhibited stable hemodynamics when VT occurred. In the meantime, with the increase in the ventricular rate, the RAP increased and the RAF decreased, while the PAF was eventually stable at a certain low level. When the ventricular rate reached 320 beats/min, VF was induced in both the models with distinct difference. In model A, VF occurred with sinus atrial rhythm, accompanied by gradual increase in the RAP and the difference between right atrial systolic and diastolic blood pressures while reductions in the LVP, PF, and PAF. However, the reductions in PF and PAF were not significant. Circulation could be sustained and there was no significant acid-base disorder. During the whole process (60 min), the right ventricular ejection was still effective (RVEF, 36%). In model B, VF occurred with AF, resulting in rapid increase in the RAP while reduction in the difference between right atrial systolic and diastolic blood pressures. Additionally, the LVP and RVP rapidly decreased to nearly 0 mmHg, while the PF and PAF also showed a significant downward trend. The circulation under this circumstance could not be sustained.

**FIGURE 4:**
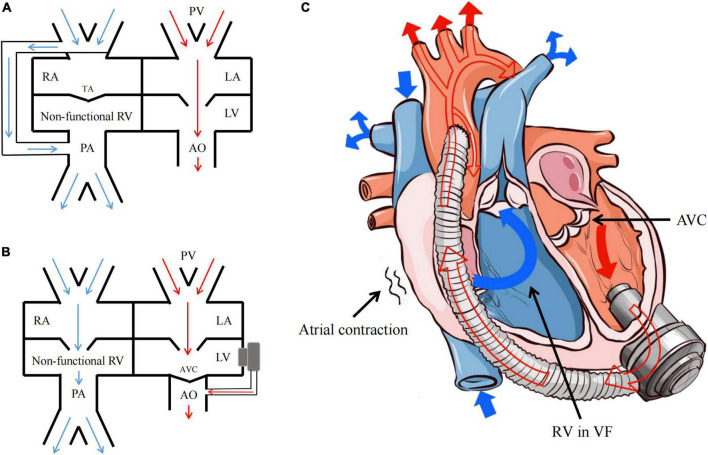
Fontan circulation and the hemodynamics of VF after CF-LVAD implantation. **(A)** Fontan circulation; **(B)** the hemodynamics of VF after CF-LVAD implantation; **(C)** the model diagram of hemodynamics in the VF state after CF-LVAD implantation. RA, right atrium; RV, right ventricle; LA, left atrium; LV, left ventricle; PA, pulmonary artery; PV, pulmonary vein; TA, tricuspid atresia; AVC, aortic valve closure.

Dib et al. (5) and Jakstaite et al. (7) considered that the hemodynamics under VF after LVAD implantation was similar to Fontan circulation through case analysis. The Fontan operation is used as a surgical strategy for functional single ventricle, with the venous blood from the superior and inferior vena cava directly drained to the pulmonary artery (Figure 4A). In that way, CVP should be equal to or greater than the pulmonary arterial pressure within a range of 12–14 mmHg. Consistently, the current comparative study also found that the hemodynamics under VF following CF-LVAD implantation resembled but was not identical to the Fontan circulation.

Firstly, under normal physiological conditions, Fontan circulation requires negative thoracic pressure. For surgical reasons, the negative thoracic pressure in the animal model was lost in the present study. As a result, the right atrium and right ventricle are not simply conduits for blood flow under VF following CF-LVAD implantation (Figure 4B). Upon ventricular systolic dysfunction, the atrial rhythm and function may be necessary for circulation maintenance after VF. Under normal circumstances, the left atrium serves as a reservoir, conduit and pump, which is responsible for about 25% left ventricular filling (11, 12). In the present study, the model A had increased amplitude of the RAP waveform after VF, which might be caused by the certain compensatory increase in the right atrial contractility according to the Frank-Starling law.

Moreover, to maintain the hemodynamics after VF following CF-LVAD implantation, RAP should be also equal to or greater than the pulmonary arterial pressure as the Fontan circulation, while the value might be lower than 12 mmHg. Different from the dependence of Fontan circulation on pulmonary vascular development, CF-LVAD implantation can reduce pulmonary vascular resistance, i.e., afterload of the right heart. In addition, the CF-LVAD will fully replace the left ventricle after VF to complete pulmonary circulation with the right atrium via cooperating with the left atrium with sinus rhythm to reduce pulmonary vascular resistance.

Combining the findings mentioned above, the present study believed that sustained atrial sinus rhythm is the hemodynamic characteristic under non-typical VF after CF-LVAD implantation. We found that the RAP and compensation in the right atrial contractility increased after blood return to the right atrium from the superior and inferior vena cava, which directly drove the blood flow from the non-functional right ventricle to the pulmonary artery and subsequently to the non-functional left ventricle through the pulmonary vein and left atrium. Finally, the blood was pumped into the systemic circulation by the CF-LVAD (Figure 4C). During the whole process, the LVP reduced due to the preload reduction of the left atrium and the complete closure of aortic valves and opening of mitral valve as result of loss of left ventricular contractility. Similar hemodynamic characteristics were found in the context of VT after CF-LVAD implantation, except for the “weak” participation of the ventricular systolic function.

Presently, the cause of VF after CF-LVAD implantation remains undetermined. Besides, routine ECG or ECG monitoring is not able to clearly illustrate the atrial rhythm under a VF condition after implantation. Hence, patients with CF-LVAD implantation might present VF with sinus atrial rhythm as described in the current study. Based on our findings and previous research (13), for those CF-LVAD-planted patients with atypical symptoms of VF, abnormal ventricular activation induced by mechanical stimulus or aberrant repolarization of the local cardiomyocytes might be a predisposing factor for VF, with the atrial rhythm and function not be affected.

There are some limitations to this study. First, the present study does not allow causal interpretation because it is an observational case study with a small sample size, although the now present findings serve as a sound basis for further mechanistic studies. Second, the VF with CF-LVAD model in this study was acute and without negative thoracic pressure, different with the chronic VF in clinical practice. However, the hemodynamics of the two types is supposed to be similar. Further larger-scale experimental studies and chronic animal experiment should be devised to explore the effect of long-term/recurrent VF on the hemodynamics and multi-organ functions.

## 5. Conclusion

The hemodynamics in the context of VF after CF-LVAD implantation resembled but was not identical to the Fontan circulation. The atrial rhythm and function might be necessary for maintenance of the pulmonary and systemic circulation under VF after implantation.

## Data availability statement

The original contributions presented in this study are included in the article/Supplementary material. Requests to access the datasets should be directed to Z-GL, liuzg@tedaich.com.

## Ethics statement

The animal study was reviewed and approved by Institutional Animal Care and Use Committee of TEDA International Cardiovascular Hospital.

## Author contributions

X-YY and J-WS: drafting article, design, data collection, data analysis, data interpretation, and critical revision of manuscript. Y-SR, Y-LC, T-WL, J-MZ, and Z-FH: concept/design, data collection, and data interpretation. Y-RZ and Z-AF: data collection. Z-GL: concept/design, data interpretation, critical revision of manuscript, and approval of manuscript. All authors contributed to the article and approved the submitted version.
