# Supplementary material for: The role of atria in ventricular fibrillation after continuous-flow left ventricular assist device implantation in ovine model
Source: Front Cardiovasc Med. 2023 Jan 9;9:1000352. doi: 10.3389/fcvm.2022.1000352 (PMC9868250; doi:10.3389/fcvm.2022.1000352)
Supplement: Supplementary file 1 [file Data_Sheet_1.docx]

Supplementary Material

# Supplementary Tables

**Supplementary** **Table 1.** The arterial blood gas baseline in the animal models after CF-LVAD implantation.

| Model | pH | PaO_2_ (mmHg) | PaCO_2_ (mmHg) | BE | HCO_3_- (mmol/L) | tHb (g/dL) | SO_2_ (%) |
| --- | --- | --- | --- | --- | --- | --- | --- |
| A | 7.62 | 405 | 34 | 12.2 | 34.1 | 11.6 | 100 |
| B | 7.54 | 208 | 28 | 2.1 | 24.0 | 10.7 | 100 |

**Supplementary Table 2.** The atrial blood gas analyses under ventricular fibrillation in Model A under different durations.

| Duration（min） | pH | PaO_2_ (mmHg) | PaCO_2_ (mmHg) | BE | HCO_3_^-^ (mmol/L) | tHb (g/dL) | SO_2_ (%) |
| --- | --- | --- | --- | --- | --- | --- | --- |
| 10 | 7.44 | 197 | 56 | 11.0 | 37.0 | 11.1 | 99 |
| 30 | 7.44 | 131 | 56 | 11.1 | 36.9 | 9.8 | 98 |
| 60 | 7.42 | 315 | 59 | 10.3 | 36.9 | 12.3 | 100 |

**Supplementary Table 3.** The atrial blood gas analyses under ventricular fibrillation in Model B.

| pH | PaO_2_ (mmHg) | PaCO_2_ (mmHg) | BE | HCO_3_^-^ (mmol/L) | tHb (g/dL) | SO_2_ (%) |
| --- | --- | --- | --- | --- | --- | --- |
| 7.40 | 70 | 47 | 2.9 | 28.2 | 11.1 | 92 |

# Supplementary Figures

**
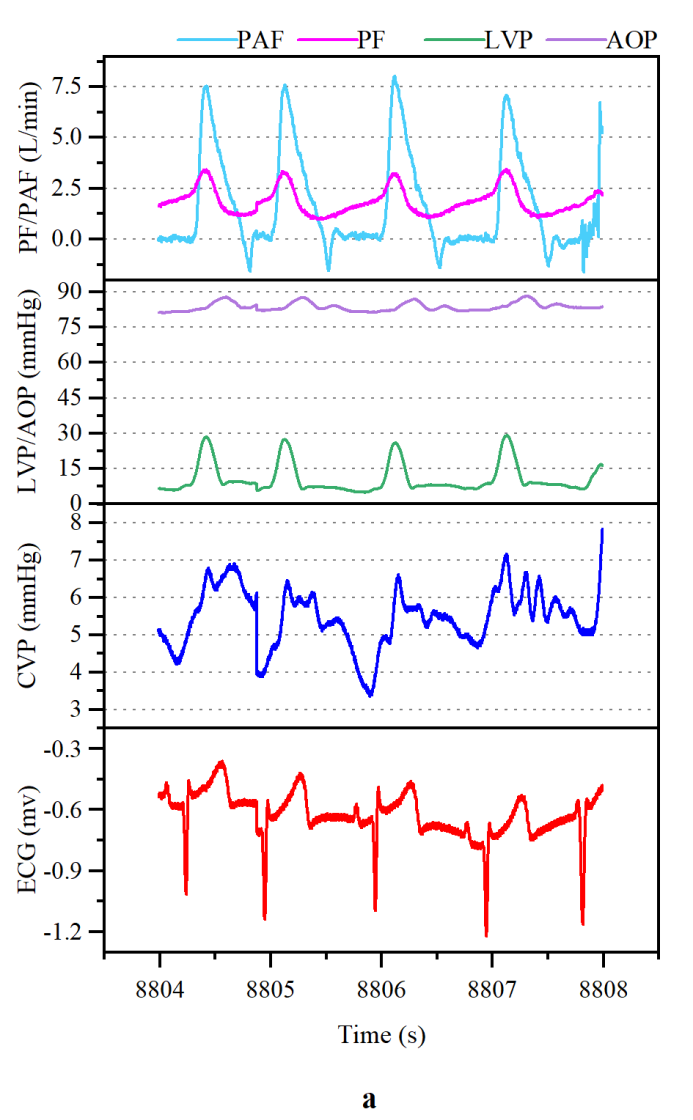
**
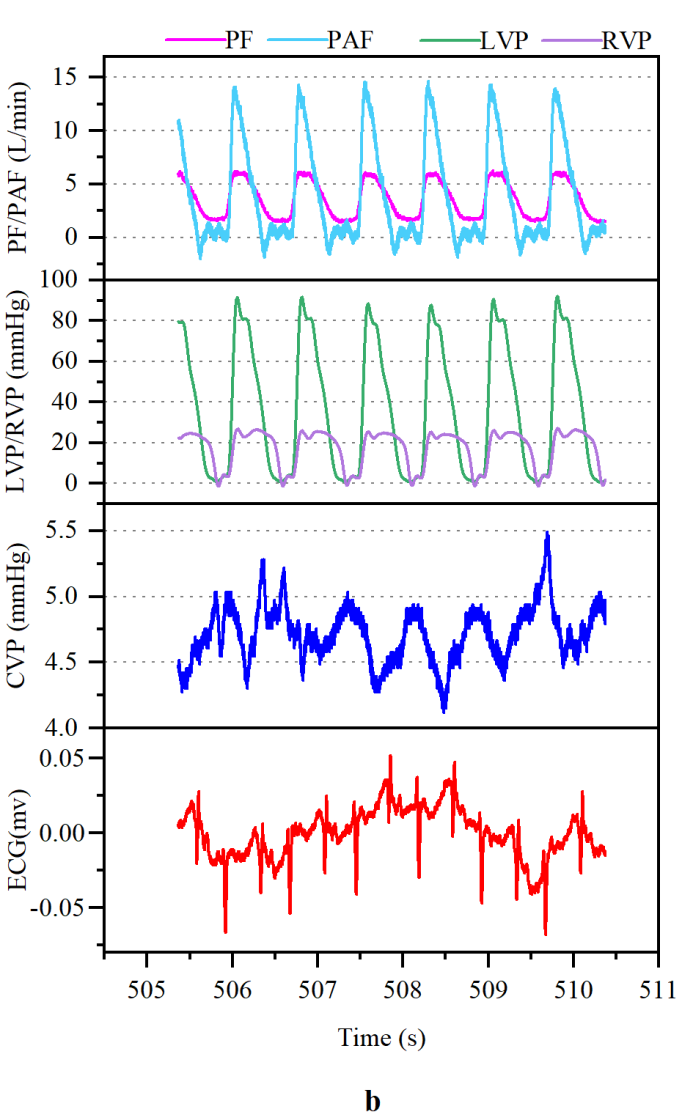


**Supplementary Figure 1.** Baseline data of the animal models with CF-LVAD implantation. a. Model A; b. Model B. PF, pump flow; PAF, pulmonary artery flow; LVP, left ventricular pressure; RVP, right ventricular pressure; LAP, left atrial pressure.

**
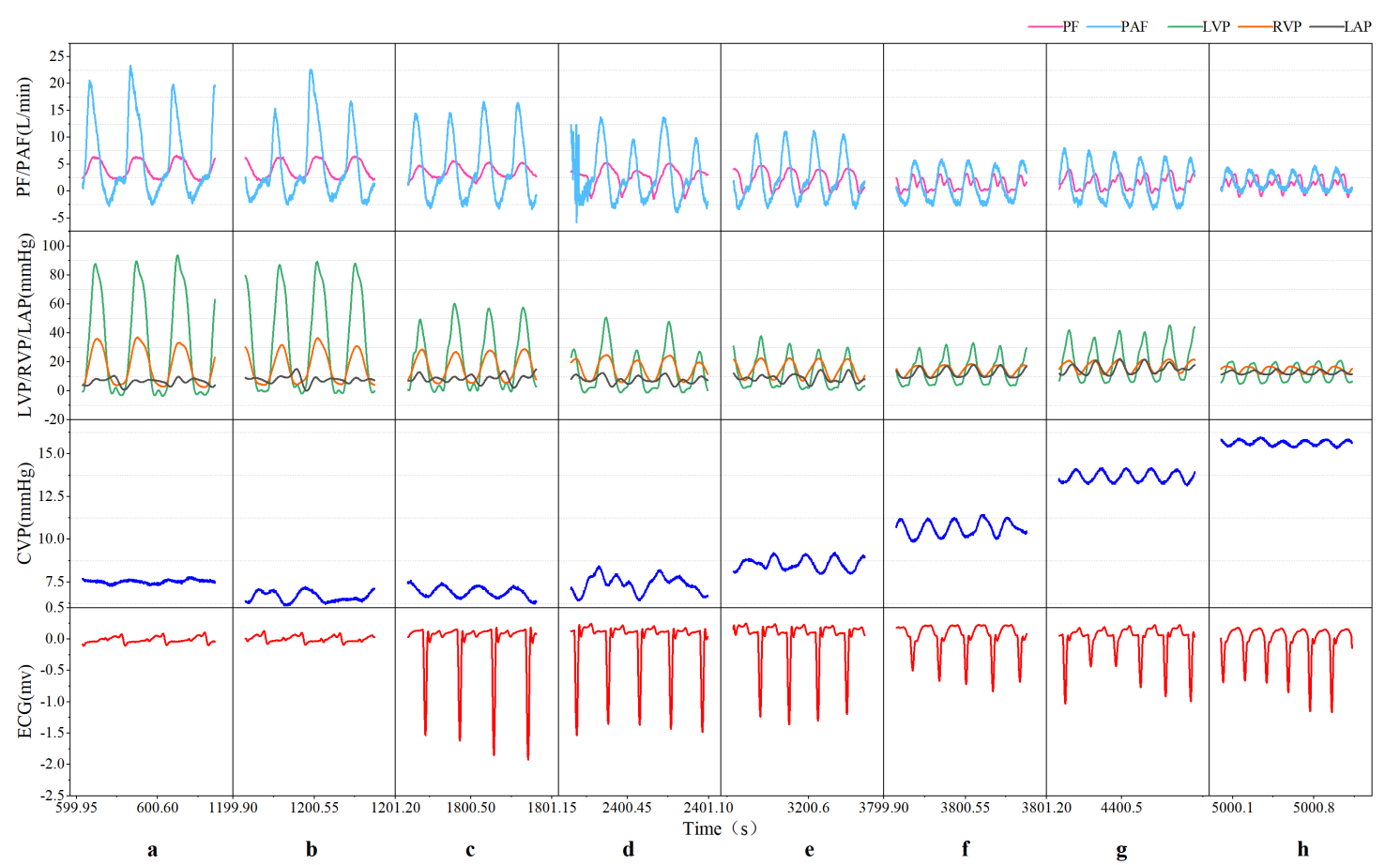
**

**Supplementary Figure 2.** VT after CF-LVAD implantation. a. ventricular rate 180 beats/min; b. ventricular rate 200 beats/min; c. ventricular rate 220 beats/min; d. ventricular rate 240 beats/min; e. ventricular rate 260 beats/min; f. ventricular rate 280 beats/min; g. ventricular rate 300 beats/min; h. ventricular rate 320 beats/min. PF, pump flow; PAF, pulmonary artery flow; LVP, left ventricular pressure; RVP, right ventricular pressure; LAP, left atrial pressure.
